# Huge Lateral Rectus Solitary Plasmacytoma Causing Shunt Extrusion

**DOI:** 10.1155/2021/5563514

**Published:** 2021-05-29

**Authors:** Nikoo Hamzeh, Mona Safizadeh, Zohreh Nozarian, Hiva Saffar, Seyed Mohsen Rafizadeh

**Affiliations:** ^1^Orbital and Oculoplastics Service, Farabi Eye Hospital, Tehran University of Medical Sciences, Tehran, Iran; ^2^Glaucoma Service, Farabi Eye Hospital, Tehran University of Medical Sciences, Tehran, Iran; ^3^Pathology Department, Farabi Eye Hospital, Tehran University of Medical Sciences, Tehran, Iran; ^4^Pathology Department, Shariati Hospital, Tehran University of Medical Sciences, Tehran, Iran

## Abstract

A 54-year-old man with a history of radiotherapy for right maxillary sinus plasmacytoma 3 years previously was referred to an orbital clinic with progressive proptosis in his right eye. His vision had deteriorated after an initial improvement after phacoemulsification 2 years before. He had undergone shunt implantation and later shunt removal following plate extrusion with the diagnosis of neovascular glaucoma following CRVO. His vision remained at no light perception afterwards, despite a controlled IOP with topical medications. In his CT scan, a large orbital mass was seen with lateral rectus involvement. He underwent deep orbitotomy for tumor resection following worsening of symptoms, and his symptoms were improved afterwards. Pathology report was consistent with plasmacytoma with anaplastic features. After tumor resection, he underwent another course of radiotherapy with complete remission of symptoms afterwards.

## 1. Introduction

Plasmacytoma is a rare tumor of small round cell or plasmacyte origin which can occur in the setting of systemic multiple myeloma or as a solitary tumor [1]. Orbital and ocular plasmacytomas are extremely rare. They may originate from the bone when involving the orbit [2] and can mimic other ocular neoplasms such as lymphoma, metastases, chondrosarcoma, or giant cell tumors [3]. Plasmacytomas of the orbit can present with diplopia, proptosis [4], and symptoms of orbital cellulitis as well. They may show unspecific imaging characteristics and may not be distinguished from other ocular tumors which would make their diagnosis more difficult [3]. Extramedullary plasmacytomas have been reported in 3% of multiple myeloma patients [2], and different treatment approaches such as excision, radiotherapy, or higher radiation doses that may damage ocular structures have been proposed [5].

## 2. Case Presentation

A 54-year-old man with a history of radiotherapy for right maxillary sinus plasmacytoma 3 years ago was referred to an orbital clinic with progressive proptosis in his right eye. His left eye had a vision of 5/10 (Snellen acuity chart), and except for a cataract, his examination was unremarkable. He had undergone uncomplicated phacoemulsification 2 years ago in his right eye with a best corrected visual acuity (BCVA) of 1/10, after which he had reported vision improvement; however, his vision had started to deteriorate at 4 months postoperatively. Initial examinations at that time did not find a specific cause for the patient's vision loss. He had failed to follow up for subsequent examinations for 1.5 years afterwards.

**Figure 1:**
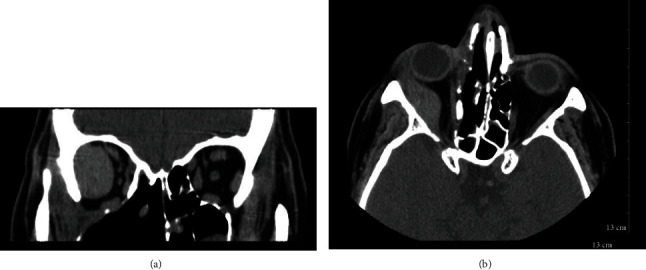
Coronal (a) and axial (b) CT scans showing large orbital mass with lateral rectus involvement.

**Figure 2:**
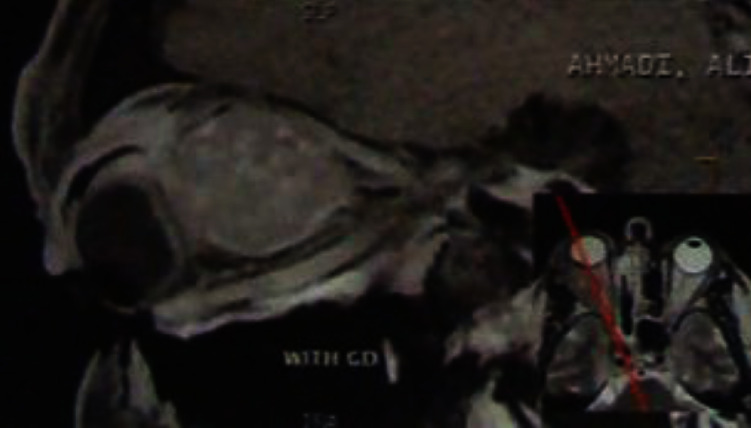
Sagittal MRI scan showing orbital mass with lateral rectus involvement.

**Figure 3:**
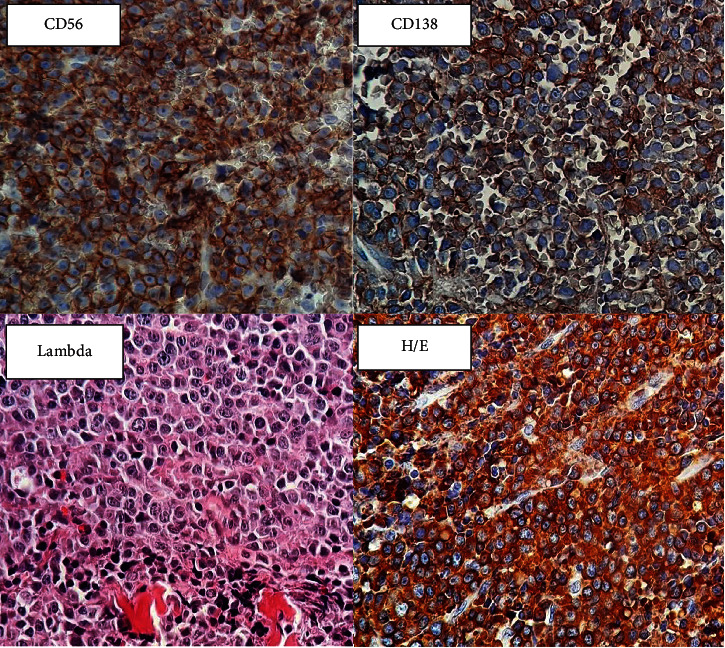
H/E-stained slide show plasmocytoid cells with infiltration of muscle fiber. (×400). Tumoral cells diffuse and strongly positive for CD138, CD56, and lambda light chain.

He was referred to a glaucoma clinic with diagnosis of uncontrolled neovascular glaucoma (NVG) secondary to central retinal vein occlusion (CRVO) about 22 months after phacoemulsification. His vision was hand motion (HM) at that time, and he had mild proptosis in his right eye. Then, he underwent Ahmed glaucoma valve (AGV) implantation with intravitreal bevacizumab (IVB) injection. Panretinal photocoagulation was done after AGV implantation. However, AGV was removed 2 weeks later due to severe proptosis and plate extrusion. He was then referred to an orbital clinic with progressive proptosis and limitation of eye movement in all gazes. He had a vision of no light perception (NLP) at the time of examination with a pale optic disc and cup to disc ratio of 10/10. His intraocular pressure (IOP) was 18 mmHg with dorzolamide, timolol, brimonidine, and latanoprost. In his CT scan, a large orbital mass was seen with lateral rectus involvement (Figures 1 and 2) and he received intravenous Solumedrol. However, he became a candidate for deep orbitotomy following worsening of symptoms and the tumor was resected and sent for pathology. After that, his symptoms were improved. The specimen processed and stained by H/E stain and showed infiltration of almost monomorphic cells with plasmacytoid features and infiltration of soft tissue and striated muscle. Immunohistochemistry examination (IHC) was done for definite diagnosis. Tumor was diffuse and strongly positive for CD138, CD56, and lambda light chain and diffuse dot-like pattern for CD99 (Figure 3). Also, the tumor was negative for LCA, CD20, CD3, PAX5, TdT, S100, CD34, kappa, CK, chromogranin, and synaptophysin, which was consistent with plasmacytoma with anaplastic features. In general, this tumor seemed to be a recurrent lesion of a previous tumor in the right maxillary sinuses.

**Figure 4:**
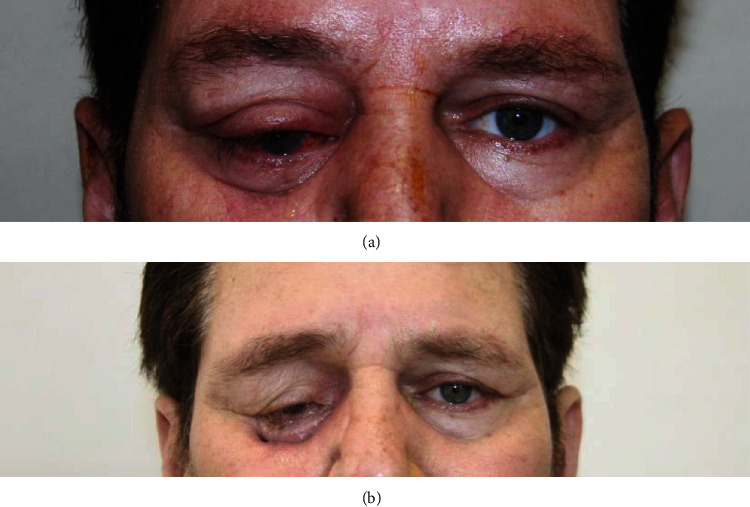
Preoperative (a) and postoperative (b) external pictures showing resolution of proptosis after orbitotomy.

After tumor resection, he underwent another course of approximately 30 sessions of radiotherapy (overall dose of 60G) for complete remission of symptoms (Figure 4). Our patient underwent a systemic work up to exclude systemic multiple myeloma, and all results including protein electrophoresis and bone marrow biopsy and aspiration were negative for systemic involvement.

## 3. Discussion

Solitary plasmacytoma is a plasma cell tumor with no evidence of systemic involvement; however, when the disease has systemic manifestations, it is called multiple myeloma. Orbital plasmacytomas can originate from the bone (solitary plasmacytoma) or from soft tissue (extramedullary) or may be a manifestation of multiple myeloma at which the tumor can be significant and produce profound vision loss [6]. They can involve the orbit, conjunctiva [7], uvea, retina, lacrimal sac [8], and ciliary body. They present as an orbital mass, epibulbar mass [9], orbital abcess [6], chalazion [6], dacryocystitis [6], granulomatous uveitis or as an isolated mass in the rectus muscles [4, 10]. An increase in the IOP or neovascular glaucoma has also been reported in the iris and ciliary body plasmacytomas [11]. Most of the reported cases of ocular plasmacytomas have associations with systemic multiple myeloma, which makes our case unique that there was no systemic involvement of the disease in our patient.

Due to the diagnosis of the maxillary sinus plasmacytoma about 3 years ago, it seems that the orbital mass was caused by a recurrence of the maxillary sinus mass. In fact, it has been stated that for the complete treatment of this aggressive tumor, both surgical resection and adequate radiotherapy are required, and a shortcoming in either of these two modalities can lead to the recurrence of this tumor. However, it is possible that inadequate resection with surgery or inadequate radiotherapy in the treatment of a previous sinus plasmacytoma has led to recurrence of the tumor in orbit [12].

Any cause of reduced venous outflow and retinal ischemia can cause CRVO [13]. Retrobulbar masses can cause compression of the retinal artery and central retinal artery occlusion (CRAO) [14]. Any vascular damage such as tumor resection can release serotonin, and acting as a vasoconstrictor, it can cause transient arterial vasospasm and a subsequent CRAO which may be managed by immediate intervention [15]; however, those scenarios are unlikely in the setting of CRVO.

CRVO is caused by compression of venules while crossing arterioles causing thrombosis, stasis, turbulence, and a resultant venous outflow obstruction. Diabetes, hypertension, hyperlipidemia, open angle glaucoma, atherosclerosis, vasculitis, dysproteinemias such as Waldenstrom macroglobulinemia and multiple myeloma, blood dyscrasias such as Polycythemia vera, and a hypercoagulable state are known risk factors for CRVO [16]. Any orbital lesion or process causing central retinal vein compression such as tumor or a carotid cavernous fistula can cause venous stasis and CRVO [13]. Retrobulbar external compression seen in thyroid eye disease or orbital masses can be accounted for CRVO as well.

In multiple myeloma, the viscosity of the venous blood flow increases due to hypercoagulopathy state, and as a result, the risk of vascular thromboembolic events, such as retinal venous occlusion, rises. Therefore, if a retinal venous occlusion occurs in the absence of common underlying diseases, one of the diseases that should probably be investigated is multiple myeloma. However, in plasmacytoma without systemic involvement, as in our case, there is no coagulation disorder and retinal vascular accident is unlikely. One possible explanation of retinal venous occlusion in this situation is the increase in intraorbital pressure as a result of the increase in the intraorbital volume due to a large tumor, which causes pressure on the central retinal vein, and consequently, venous turbulence and venous thrombosis may develop [13, 16].

Since our patient showed no evidence of systemic multiple myeloma and had none of the risk factors of CRVO, It is not clear whether his development of CRVO and subsequent NVG was due to ischemia because of previous radiotherapy or ischemic and compressive effects of the plasmacytoma; however, since he had an acceptable vision following radiotherapy for a few years and his vision improved after cataract surgery and only started to worsen few months later with the onset of proptosis, we can propose that the orbital lesion might have been a contributing factor to the development of CRVO.

## Data Availability

All the data and information regarding the patient can be accessed in Farabi Hospital files, after contact with the corresponding author.
